# Unravelling the Interplay: Exploring the Influence of Previous Hepatitis B Virus, Hepatitis A Virus, and Hepatitis E Virus Infections on Non-alcoholic Fatty Liver Disease

**DOI:** 10.7759/cureus.44969

**Published:** 2023-09-09

**Authors:** Vinay Narladkar, Aman Agrawal, Sanket S Bakshi, Swarupa Chakole, Aniket G Pathade, Seema Yelne

**Affiliations:** 1 Medicine, Jawaharlal Nehru Medical College, Datta Meghe Institute of Higher Education and Research, Wardha, IND; 2 Community Medicine, Jawaharlal Nehru Medical College, Datta Meghe Institute of Higher Education and Research, Wardha, IND; 3 Research and Development, Jawaharlal Nehru Medical College, Datta Meghe Institute of Higher Education and Research, Wardha, IND; 4 Nursing, Shalinitai Meghe College of Nursing, Datta Meghe Institute of Higher Education and Research, Wardha, IND

**Keywords:** treatment, diagnosis, mechanisms, interplay, nafld, viral infections

## Abstract

The intricate interplay between viral infections and non-alcoholic fatty liver disease (NAFLD) presents a fascinating and clinically significant intersection of virology and hepatology. This review article delves into the complex relationship between hepatitis B virus (HBV), hepatitis A virus (HAV), hepatitis E virus (HEV), and NAFLD. It outlines the shared mechanisms linking viral infections to NAFLD development, including their effects on lipid metabolism, immune responses, inflammation, and gut microbiota. The clinical implications of this interplay are explored, including challenges in diagnosis and management and potential therapeutic strategies. The review emphasises the need for a comprehensive understanding of these interactions as they impact disease progression, risk stratification, and treatment decisions. Furthermore, it highlights the importance of integrated approaches and personalised treatment paradigms for optimising patient care. As we navigate this intricate crossroads, the insights gained can reshape our understanding of liver health and contribute to more effective strategies for managing viral infections and NAFLD.

## Introduction and background

Non-alcoholic fatty liver disease (NAFLD) stands as one of the most prevalent chronic liver disorders worldwide, encompassing a spectrum of liver conditions ranging from simple steatosis to non-alcoholic steatohepatitis (NASH) and eventually progressing to cirrhosis and hepatocellular carcinoma. The surge in NAFLD's prevalence is closely intertwined with the global epidemic of obesity, sedentary lifestyles, and unhealthy dietary habits. As NAFLD rises, its implications for public health, healthcare systems, and patient well-being become increasingly evident [1,2].

The intricate relationship between viral infections and NAFLD has emerged as a fascinating and clinically significant area of research. Hepatitis B virus (HBV), hepatitis A virus (HAV), and hepatitis E virus (HEV) infections each carry their distinct impact on liver health. In this context, unravelling the interplay between previous HBV, HAV, and HEV infections and their potential influence on NAFLD presents a compelling avenue for investigation. Understanding how these viral infections intersect with NAFLD development can provide valuable insights into disease pathogenesis, prognosis, and potential therapeutic strategies [3,4].

The intricate interplay between viral infections and NAFLD is multifaceted. Viral infections can trigger intricate immunological responses and perturbations in lipid metabolism, both of which are implicated in the development and progression of NAFLD. Chronic inflammation triggered by viral infections can contribute to hepatocyte injury, initiating a cascade of events that lead to hepatic steatosis, oxidative stress, and fibrogenesis. Additionally, viral infections may modulate gut microbiota composition, influencing metabolic pathways associated with NAFLD [5,6].

HBV, HAV, and HEV infections are the most common viral infections affecting the liver. HBV infection, a primary global health concern, has been extensively studied for its role in liver disease, ranging from acute hepatitis to chronic infection, cirrhosis, and hepatocellular carcinoma. HAV, typically transmitted through the fecal-oral route, is a significant cause of acute viral hepatitis. HEV, primarily transmitted through contaminated water and food, can result in acute or chronic hepatitis, especially in specific geographic regions [7].

The primary objective of this review article was to provide a comprehensive understanding of the potential influence of previous HBV, HAV, and HEV infections on the development, progression, and clinical outcomes of NAFLD. This review aims to shed light on the complex interplay between these viral infections and NAFLD by synthesizing existing literature and exploring the underlying mechanisms. Moreover, the article will discuss the clinical implications of this interplay, including potential challenges in diagnosis and management. Additionally, the review will identify research gaps and suggest avenues for future investigation, aiming to contribute to advancing knowledge in this vital field at the intersection of virology and hepatology.

## Review

HBV infection and NAFLD

Overview of HBV Infection and Its Impact on the Liver

HBV infection stands as a significant global health challenge, affecting an estimated 250 million individuals worldwide. This virus profoundly impacts liver health, primarily targeting hepatocytes, which are critical functional cells of the liver. The consequence of the interaction of HBV with hepatocytes spans a diverse range of liver-related diseases, encompassing acute and chronic hepatitis, cirrhosis, and even the development of hepatocellular carcinoma (HCC), a form of liver cancer. The clinical outcomes of HBV infection exhibit notable heterogeneity, with disease progression varying widely among affected individuals [8].

HBV infection is characterized by its ability to establish chronicity, with a subset of infected individuals transitioning from acute to chronic infection. During chronic HBV infection, an intricate interplay of immune responses and molecular pathways unfolds within the liver. These responses can profoundly disrupt liver homeostasis, contributing to inflammation, tissue damage, and, ultimately, the development of liver diseases [9].

One significant avenue through which chronic HBV infection influences liver health is its impact on immune responses. The virus stimulates the immune system, resulting in persistent inflammation within the liver. This ongoing inflammatory milieu can lead to hepatocyte injury and the release of pro-inflammatory molecules, further propagating liver damage. Significantly, these immune disturbances are not limited to the liver; they can also have systemic effects contributing to metabolic imbalances [10].

Moreover, chronic HBV infection exerts intricate effects on crucial molecular pathways governing lipid metabolism and energy equilibrium within hepatocytes. This intricate interplay between viral factors and host cellular mechanisms can disrupt the delicate balance of lipid homeostasis, culminating in the intracellular accumulation of lipids, an essential characteristic of NAFLD. This cascade of events underscores how chronic HBV infection can create an environment ripe for perturbations in both the liver's immune responses and metabolic functions, potentially acting synergistically to fuel the emergence and progression of NAFLD [11].

The convergence of chronic HBV infection with intricate metabolic pathways underscores its capability to influence lipid metabolism profoundly. By perturbing the regulatory networks that oversee lipid storage, utilization, and synthesis, the virus can instigate aberrant lipid accumulation within hepatocytes. Such a disruption in lipid homeostasis not only shapes the cellular milieu but also sets the stage for developing hepatic steatosis, a fundamental component of NAFLD. However, the implications of chronic HBV infection extend beyond mere metabolic derangements [11].

This infectious milieu can significantly impact the liver's immune landscape, orchestrating a complex interplay between viral persistence and host immune responses. The virus-induced alterations in the immune microenvironment can undermine the liver's ability to mount effective immune surveillance and antiviral defence. Consequently, this dysregulated immune milieu may facilitate the progression of NAFLD, contributing to the transition from simple hepatic steatosis to more severe stages of liver disease. Chronic HBV infection operates as a multifaceted disruptor, perturbing both metabolic processes and immune responses within the liver. This dual impact sheds light on the intricate interactions underlying NAFLD pathogenesis. It underscores the need for a comprehensive understanding of the virus-host interplay in the context of liver diseases. By unravelling the intricate connections between viral factors, metabolic pathways, and immune responses, we can potentially identify novel avenues for therapeutic intervention in NAFLD and related disorders [11].

Mechanisms Linking HBV Infection to NAFLD Development

The relationship between chronic hepatitis B virus (HBV) infection and the development of non-alcoholic fatty liver disease (NAFLD) is intricate and characterized by the convergence of multiple interconnected mechanisms. These mechanisms collectively contribute to hepatocyte injury and metabolic dysregulation, ultimately influencing the progression of NAFLD [12].

Viral proteins and their effects on lipid metabolism: HBV, as a master manipulator of cellular processes, encodes various viral proteins, among which the hepatitis B virus X protein (HBx) plays a pivotal role. HBx can directly influence lipid metabolism within hepatocytes. It can impact critical aspects of lipid homeostasis, including lipid uptake, synthesis, and storage. Through its interactions with cellular machinery, HBx can disturb the equilibrium between lipid synthesis and breakdown, potentially leading to an accumulation of lipids within hepatocytes. This aberrant lipid accumulation, known as hepatic steatosis, is a fundamental hallmark of NAFLD development [13].

Immune response modulation and inflammation: Chronic HBV infection triggers a persistent immune response as the host attempts to combat the virus. However, this prolonged immune activation can have unintended consequences for liver health. The sustained presence of viral antigens and inflammatory signals leads to chronic inflammation within the liver. This inflammatory environment contributes to hepatocyte injury and the release of pro-inflammatory cytokines. Significantly, chronic inflammation can disrupt normal lipid homeostasis within hepatocytes, leading to an imbalance between lipid uptake, synthesis, and clearance. As a result, lipids accumulate within hepatocytes, promoting the formation of lipid droplets and the development of hepatic steatosis [14].

HAV infection and NAFLD

Introduction to HAV Infection and Its Effects on the Liver

Hepatitis A (HAV) infection is recognized as a prevalent etiological agent responsible for acute viral hepatitis. This infection is primarily transmitted through the fecal-oral route, often from contaminated water or food sources. Traditionally, HAV infection is characterized by an acute onset of inflammation within the liver, which can lead to symptoms like jaundice, fatigue, and abdominal discomfort. However, recent insights have unveiled a more intricate picture of the relationship between HAV infection and liver health [15].

Although HAV infection's acute phase is well defined, emerging evidence suggests its impact on the liver might extend beyond this initial stage. While acute inflammation is the hallmark, researchers have explored the possibility that HAV infection's effects on liver health could persist or even evolve into more complex outcomes. This realization is shifting our understanding of the broader implications of HAV infection beyond the acute setting [16].

The potential connection between HAV infection and the development and progression of non-alcoholic fatty liver disease (NAFLD) is particularly interesting. NAFLD, characterized by fat accumulation within the liver cells in individuals with minimal or no alcohol consumption, has become a significant global health concern. Interestingly, studies have indicated that HAV infection might influence metabolic pathways and immune responses that intersect with NAFLD pathogenesis. This raises intriguing questions about whether the acute phase of HAV infection could set the stage for metabolic alterations and inflammatory responses contributing to developing NAFLD in the longer term [17].

While our understanding of the precise mechanisms underlying the relationship between HAV infection and NAFLD is still evolving, the expanding body of research underscores the need for a more comprehensive assessment of the consequences of HAV infection on liver health. Recognizing the potential for broader effects beyond the acute phase, healthcare practitioners and researchers are prompted to explore the intricate interplay between HAV infection, metabolic disturbances, and NAFLD pathogenesis. This exploration has the potential to offer novel insights into liver disease dynamics and pave the way for more holistic strategies for diagnosing, managing, and ultimately preventing liver-related complications in individuals with a history of HAV infection [18].

Potential Mechanisms Connecting HAV Infection to NAFLD

Immune response modulation and liver inflammation: HAV infection triggers a vigorous immune response to clear the virus from the body. This immune activation involves the release of various cytokines and immune cells. Importantly, this immune response is not limited to the site of infection, as signals from the infected tissues can be transmitted to distant organs, including the liver. Consequently, the immune activation associated with HAV infection may extend to the liver, potentially leading to localized inflammation [19]. The inflammatory response within the liver can disrupt its normal metabolic functions. Inflammation is known to interfere with insulin signalling and promote insulin resistance, a hallmark of NAFLD. Additionally, inflammatory mediators can impact lipid metabolism, impairing the breakdown and utilization of fats. This metabolic disturbance can lead to the accumulation of triglycerides within hepatocytes, contributing to hepatic steatosis, the initial stage of NAFLD [20].

Altered gut microbiota and immune-metabolic interplay: The gut microbiota, a complex community of microorganisms inhabiting the digestive tract, exerts a profound influence on various physiological processes, including metabolism and immune responses. Recent insights underscore the significance of gut microbiota dysbiosis in the pathogenesis of NAFLD. Intriguingly, there is growing evidence suggesting a potential interplay between HAV infection and alterations in gut microbiota composition, although the precise underlying mechanisms remain speculative [21]. This intricate connection highlights the gut microbiome's multifaceted role in influencing metabolic changes and immune responses, contributing to the intricate landscape of NAFLD development.

Alterations in the gut microbiota induced by HAV infection can potentially perturb the delicate equilibrium between beneficial and pathogenic microorganisms within the gut. This disruption in the gut microbiota, known as dysbiosis, can potentially impact vital processes such as nutrient breakdown and absorption, consequently influencing energy metabolism. It's important to note that specific strains of gut bacteria play a role in producing essential metabolites like short-chain fatty acids, which actively regulate various metabolic pathways. These intricate interactions between the gut microbiota and metabolic processes hold significant implications. Disturbances in these interactions could potentially render individuals more susceptible to metabolic shifts that contribute to conditions such as NAFLD [22]. It is worth highlighting that dysbiosis might also exert broader effects on the immune system beyond its immediate impact on nutrient metabolism. The gut microbiome plays a pivotal role in educating and modulating immune responses. Thus, disruptions in the microbiome could potentially lead to immune imbalances that further exacerbate metabolic irregularities. Considering the interconnected nature of these factors, a comprehensive understanding of how HAV infection-associated gut microbiota changes influence metabolic pathways and immune responses can provide valuable insights into the development of conditions like NAFLD. Further research is warranted to unravel the intricate mechanisms underpinning these relationships and their potential therapeutic implications.

Relevant Studies and Data Supporting the Association

The potential connection between HAV infection and NAFLD has garnered attention. These investigations have primarily concentrated on understanding how HAV infection might influence liver health, particularly in the aftermath of an acute HAV infection. The main focus of these studies has been to assess whether acute HAV infection plays a role in developing hepatic steatosis, a hallmark of NAFLD characterized by fat accumulation within liver cells [23].

The outcomes of these studies have been diverse, with varying results reported. Some studies have indicated a notable increase in hepatic steatosis among individuals recently recovering from an acute HAV infection. This suggests that there might be a link between HAV infection and the propensity for fat to accumulate within the liver. However, it is important to note that the relationship isn't uniform across all studies, and some investigations have yet to observe a substantial association between acute HAV infection and hepatic steatosis [24].

Additionally, researchers have conducted longitudinal studies that track individuals with a history of HAV infection over time. These studies have revealed potential changes in metabolic parameters that could contribute to the development of NAFLD. Alterations in insulin sensitivity, lipid profile disturbances, and other metabolic markers fluctuations have been observed among individuals with a history of HAV infection. These changes suggest that HAV infection might influence metabolic processes in a way that could predispose individuals to developing NAFLD [25].

Clinical implications and management

Discussion of the Clinical Relevance of the Interplay Between Viral Infections and NAFLD

Disease progression: The convergence of viral infections and NAFLD acts as a catalyst for disease progression. Viral infections, acting in synergy with the metabolic disruptions intrinsic to NAFLD, can accelerate the transition from benign hepatic steatosis to more severe conditions such as non-alcoholic steatohepatitis (NASH) and fibrosis. The intricate interplay between viral factors and the underlying metabolic perturbations can amplify the inflammatory cascade, hastening the progression toward fibrogenesis and more advanced liver pathologies [20].

Diagnostic challenges: The intricate interplay between viral infections and NAFLD presents diagnostic complexities. Viral infections might obfuscate or mimic the clinical presentation of NAFLD, creating challenges in accurate diagnosis. Discerning the effects of viral hepatitis and those of NAFLD is pivotal to initiating the right therapeutic strategies and tailored patient management [26].

Therapeutic considerations: The coexistence of viral infections and NAFLD introduces intricate therapeutic considerations. In some cases, medications designed for NAFLD management might inadvertently interact with antiviral therapies, potentially impacting viral replication or efficacy. Conversely, the effects of antiviral treatments could influence the metabolic milieu relevant to NAFLD, necessitating meticulous evaluation to prevent unintended consequences [27].

Challenges in Diagnosis and Management of Patients With Both Infections and NAFLD

Differential diagnosis: Distinguishing between liver injury caused by viral infections, such as HBV, HAV, and HEV, and that caused by NAFLD can be a daunting task due to the convergence of clinical and biochemical features. Both conditions may manifest with elevated liver enzymes, fatigue, and even jaundice, creating an intricate diagnostic landscape. Discerning the underlying aetiology becomes paramount to tailoring appropriate treatment strategies [3].

Assessing disease severity: Viral infections and NAFLD may intersect in a manner that complicates the assessment of disease severity. Viral infections can obscure the clinical indicators of NAFLD, masking the true extent of liver damage. Conversely, advanced fibrosis due to viral infections might overshadow the accurate staging of NAFLD-related fibrosis. This overlapping complexity makes accurate disease stratification challenging, potentially leading to underestimation or misclassification of disease severity [28].

Treatment decision dilemma: When faced with patients harbouring viral infections and NAFLD, selecting appropriate treatment strategies becomes a multifaceted dilemma. The intricate balance between antiviral therapy and NAFLD management necessitates a personalized approach considering the specific viral agent, the severity of liver involvement, and potential drug interactions. Achieving optimal therapeutic outcomes while mitigating the risk of adverse events requires a thorough understanding of each condition's nuances and potential impacts on treatment efficacy [29].

Potential Therapeutic Strategies Targeting Both Viral Infections and NAFLD

Lifestyle adaptations: Lifestyle interventions encompassing dietary adjustments and physical exercise exhibit considerable potential as fundamental approaches in managing viral infections and NAFLD. While the central objective in addressing NAFLD is to ameliorate metabolic indicators and diminish the buildup of hepatic lipids, these interventions possess the capacity for more extensive influence. Consistent engagement in physical activity and the adoption of a well-balanced dietary regimen hold the capacity to augment immune functionality, a pivotal factor in effectively countering viral infections. Moreover, the benefits extend further: weight reduction and enhanced insulin sensitivity, attainable through these lifestyle adaptations, can positively influence viral replication and inflammation. This, in turn, contributes to a more effective management strategy for both sets of conditions [30].

Combined therapies: Investigating therapies that concurrently target viral infections and NAFLD represents an innovative avenue for patient care. Drugs with dual mechanisms of action could offer substantial benefits. For instance, antiviral agents with positive metabolic effects, such as reducing insulin resistance or inflammation, could simultaneously address viral infections and the underlying metabolic disruptions in NAFLD. These dual-action drugs mitigate the need for separate treatment regimens, simplifying management and enhancing patient adherence [31].

Personalized approaches: Tailoring treatment plans based on the individual's unique circumstances offers a nuanced strategy to optimize outcomes in viral infections and NAFLD. Considering the intricate interplay between these conditions, personalized approaches account for the patient's specific viral load, viral subtype, genetic predisposition, and metabolic profile. Such an approach allows clinicians to navigate potential drug interactions, synergies, and contraindications effectively, crafting treatment plans that simultaneously address the complexities of both conditions. This tailored strategy also aligns with the broader trend of precision medicine, ensuring that therapeutic interventions are finely tuned to each patient's needs [32].

The interplay between viral infections and NAFLD

Crossroads Between Viral Infections and NAFLD Pathogenesis

The concept of the "crossroads between viral infections and NAFLD pathogenesis" captures the pivotal juncture where viral infections, such as HBV, HAV, and HEV, intersect with the complex landscape of non-alcoholic fatty liver disease (NAFLD) pathogenesis. This metaphorical crossroads represents a dynamic convergence of two distinct domains: virology and hepatology. In this intricate interplay, virology, which explores the behaviour and impact of viruses, meets hepatology, which delves into the study of the liver and its associated disorders [9].

At this juncture, the interaction between viral infections and NAFLD unfolds, holding immense potential to influence the trajectory of NAFLD's development and progression. This interaction is not one-dimensional; it encompasses multifaceted relationships that encompass molecular, immunological, and metabolic mechanisms. These interactions, at the "crossroads," can contribute to a complex interplay, shaping the overall health of the liver and influencing disease outcomes [33].

Shared Mechanisms and Pathways Influencing Disease Progression

Inflammation and oxidative stress: Both viral infections and NAFLD trigger inflammatory responses and oxidative stress within the liver. These stressors, originating from viral agents and metabolic disturbances, can synergistically contribute to hepatocyte injury. The cumulative effect of these stressors may play a pivotal role in propelling the transition from an initial state of simple steatosis to more advanced stages of liver disease, including non-alcoholic steatohepatitis (NASH) and fibrosis [34].

Immune response dysregulation: Chronic viral infections often induce immune response dysregulation, marked by persistent inflammation and immune exhaustion. This compromised immune state can create an environment conducive to exacerbating inflammatory processes seen in NAFLD. The interplay between viral infections and NAFLD-driven inflammation might lead to a positive feedback loop, where immune disturbances in one condition further intensify inflammatory cascades in the other. This heightened inflammatory milieu can accelerate the development of fibrosis, a hallmark of advanced NAFLD [35].

Lipid metabolism: Both viral infections and NAFLD influence lipid metabolism, contributing to hepatic lipid accumulation. Viral factors and immune responses can perturb various facets of lipid handling within hepatocytes. For instance, these factors can enhance lipid uptake, alter lipid synthesis, and impact the export of lipids from hepatocytes. Such disruptions synergistically amplify the metabolic perturbations that characterize NAFLD, fostering an environment conducive to accumulating intracellular lipids [36].

Possible Synergistic Effects of Multiple Infections on NAFLD Development

Viral load and severity: The cumulative viral load may intensify when different viral infections coexist within the liver. This increased viral burden could lead to a heightened inflammatory response, exacerbating NAFLD's metabolic disturbances. The liver, already challenged by multiple viral insults, might experience amplified oxidative stress, inflammation, and hepatocyte injury. Consequently, the combined impact of these factors might contribute to more severe disease outcomes and hasten the progression of NAFLD [37].

Immune responses: The immune system's response to viral infections is complex and interconnected. When multiple viral infections occur, the interactions between immune responses can result in intricate dynamics. The cross-reactivity between immune responses targeting different viruses might trigger a cascade of events that influence the inflammatory milieu within the liver. This could lead to an amplified immune response that impacts liver inflammation, fostering an environment conducive to disease progression [38].

Metabolic burden: Viral infections can impose a substantial metabolic burden on the liver. These infections can alter metabolic pathways related to lipid metabolism, glucose regulation, and mitochondrial function. In the context of NAFLD, which already involves metabolic disturbances, the additional burden of viral infections can create a synergistic effect. The combination of disrupted metabolic processes from viral infections and NAFLD might amplify the underlying metabolic perturbations, contributing to more severe liver pathology [39].

Clinical implications and management

Disease Progression: Accelerated Pathways

The convergence of viral infections and NAFLD manifests in an intricate interaction that amplifies the trajectory of liver disease progression. This interplay can accelerate the onset and advancement of liver pathology, potentially leading to grave complications such as cirrhosis and hepatocellular carcinoma. By synergistically exacerbating inflammation, oxidative stress, and metabolic perturbations, this interplay magnifies the severity of liver pathophysiology, ultimately impacting the long-term prognosis of affected individuals [40].

Diagnostic Challenges: Navigating Complexities

Accurately diagnosing patients who harbour viral infections and NAFLD poses a formidable challenge due to the intricate overlap in clinical features and laboratory findings. Distinguishing between the distinct contributions of each condition becomes pivotal for designing an effective treatment strategy. The complex interplay between these entities can mask or mimic specific diagnostic markers, necessitating astute differentiation to unravel the underlying pathologies accurately [28].

Treatment Complexity: Balancing Dual Concerns

The dual presence of viral infections and NAFLD calls for a nuanced therapeutic approach that carefully balances managing both conditions. The potential interactions between antiviral medications and NAFLD therapies underscore the necessity for meticulous planning to avert unintended consequences. Striking the delicate balance between addressing viral replication and mitigating metabolic disturbances is paramount to ensuring optimal treatment efficacy and minimizing potential adverse effects [41].

Challenges in Diagnosis and Management of Patients With Both Infections and NAFLD

Differential diagnosis: Distinguishing between the specific effects of viral infections and those attributed to NAFLD-related liver damage requires a comprehensive evaluation of various factors. This includes meticulously analyzing the patient's clinical history and integrating serological markers and imaging studies. The challenge lies in accurately attributing observed abnormalities to their origins, as the clinical presentations of viral infections and NAFLD can exhibit striking similarities [28].

Risk assessment: Accurately assessing the severity and progression of liver disease becomes particularly complex when viral infections and NAFLD are concurrently present. Understanding the individual contribution of each factor to liver injury is paramount for appropriate risk stratification. This requires a nuanced analysis of factors such as fibrosis stage, inflammation markers, and metabolic parameters. Distinguish between these contributors to avoid misinterpreting disease progression and suboptimal patient management [42].

Treatment decision complexity: When viral infections and NAFLD coexist, selecting an optimal therapeutic approach becomes a delicate balancing act. Antiviral agents that treat viral infections may interact with NAFLD-specific treatments, potentially altering treatment efficacy or safety profiles. Developing tailored treatment plans requires deeply understanding potential interactions and carefully considering the patient's condition. Individualized care plans must account for these complexities to ensure optimal therapeutic outcomes [43].

Potential Therapeutic Strategies Targeting Both Viral Infections and NAFLD

Lifestyle modifications: Lifestyle interventions are pivotal in managing viral infections and NAFLD. Dietary modifications, including reducing excessive sugar and saturated fat intake while increasing the consumption of nutrient-rich foods, benefit both conditions. Incorporating regular exercise into daily routines facilitates weight loss and improves insulin sensitivity and cardiovascular well-being. These collective adjustments contribute to managing metabolic parameters, mitigating inflammation, and reinforcing immune function. As a result, both viral infections and NAFLD can experience positive effects [44].

Combined therapies: Exploring therapeutic agents with dual mechanisms of action is a promising avenue for addressing the complex interplay between viral infections and NAFLD. Agents that simultaneously target viral replication, inflammation, and metabolic factors could mitigate the adverse effects of both conditions. This integrated therapeutic approach would require a comprehensive understanding of the molecular pathways underlying both viral infections and NAFLD, enabling the development of treatments that effectively address the intertwined challenges posed by these conditions [45].

Tailored management: Personalized treatment plans considering each patient's unique characteristics are crucial when managing viral infections and NAFLD. Tailored management involves assessing the patient's specific condition, viral load, metabolic profile, and potential interactions between different therapies. Multidisciplinary collaboration among hepatologists, infectious disease specialists, and endocrinologists is essential to formulate a comprehensive treatment strategy that optimizes therapeutic outcomes and minimizes risks [46].

Future directions and research gaps

Identifying Gaps in Current Knowledge and Understanding

Mechanistic insights: The intricate molecular underpinnings governing the interactions between viral infections and NAFLD remain largely obscure. A more comprehensive exploration of these mechanisms is essential to show how viral components intricately influence critical processes such as lipid metabolism, immune responses, and inflammation within the context of NAFLD. Unravelling these intricate connections could elucidate pivotal points for intervention and therapeutic targeting.

Longitudinal studies: While existing research has provided valuable insights into the immediate effects of viral infections on NAFLD, a critical need remains for in-depth longitudinal studies encompassing extended periods. These studies would enable a nuanced understanding of the prolonged impact of viral infections on the progression and outcomes of NAFLD over time. Furthermore, unravelling the temporal relationship between viral infection and the development of NAFLD is paramount for uncovering the sequence of events and potential causal links.

Synergistic effects: The potential additive or synergistic effects arising from the coexistence of multiple viral infections on NAFLD pathogenesis are an area that requires further exploration. Limited research has addressed how various viruses interact and potentially amplify the complex processes underlying NAFLD progression. These potential synergies could offer insights into unique disease trajectories and novel therapeutic avenues.

Suggesting Areas for Future Research and Investigation

Molecular mechanisms: A comprehensive understanding of the intricate molecular connections between viral infections and NAFLD is essential. This entails a thorough investigation into the interactions between viral proteins and host cellular machinery, contributing to the complex events that culminate in hepatic lipid accumulation, inflammation, and fibrosis. Through a detailed elucidation of these precise interactions and pathways, researchers can identify innovative therapeutic targets and potential strategies for intervention. Such insights hold the promise of novel drugs capable of addressing the dual impact of viral infections and NAFLD, potentially mitigating the adverse outcomes of this combined burden.

Immunological interactions: The dynamic crosstalk between viral infections and NAFLD on an immunological level remains a significant research frontier. Understanding how viral-induced immune responses modulate the inflammatory milieu within the liver is critical. This exploration extends beyond isolated pathways to investigate the broader systemic and localized immunological responses triggered by viral infections and subsequently influence the development and progression of NAFLD. Insights into these complex immunological interactions could lead to innovative therapies targeting the inflammatory component of viral infections and NAFLD.

Clinical outcomes: To comprehensively comprehend the impact of viral infections on NAFLD-related clinical outcomes, prospective studies are essential. Longitudinal investigations should examine the trajectory of disease progression, the potential for fibrosis regression, and the response to treatment interventions in individuals affected by viral infections and NAFLD. These studies will shed light on the overall prognosis of this complex coexistence and provide valuable data that can guide clinical management decisions, helping to tailor personalized treatment strategies for this unique patient population.

Importance of Exploring Preventive Measures and Personalized Treatment Approaches

Preventive measures: The exploration of preventive measures takes on paramount importance. This involves thoroughly investigating interventions to mitigate the risk of NAFLD development in individuals who have previously encountered viral infections. By uncovering ways to delay or prevent the onset of NAFLD within this subset of patients, the potential to significantly reduce the overall disease burden emerges.

Precision medicine: The concept of precision medicine emerges as a pivotal approach in managing individuals grappling with viral infections and NAFLD. This entails the development of tailored treatment strategies that meticulously consider each patient's unique virological and metabolic profiles. Acknowledging the distinct characteristics of both conditions, this approach endeavours to optimise therapeutic outcomes while minimising any potential interactions between antiviral agents and treatments specific to NAFLD.

Risk stratification: A critical aspect of effective management involves the identification of reliable biomarkers and clinical parameters that facilitate accurate risk stratification in individuals affected by viral infections. The goal is to empower clinicians to differentiate the varying degrees of risk and vulnerability among patients. This knowledge then guides the formulation of tailored surveillance and management plans, ensuring that each patient's needs are met with precision and appropriateness.

## Conclusions

In conclusion, the intricate interplay between viral infections such as HBV, HAV, and HEV, and the development of NAFLD, represents a complex phenomenon that spans the fields of virology and hepatology. This review has shed light on the interconnected pathways and mechanisms through which viral infections can exacerbate NAFLD's metabolic disruptions and inflammatory processes. As we navigate the intersection of these two domains, it becomes abundantly clear that this interplay extends beyond mere academic curiosity, emerging as a pivotal determinant of disease progression, diagnostic complexities, and therapeutic strategies for individuals grappling with viral infections and NAFLD. The significance of this intricate relationship resonates far beyond the realm of medical research, casting an influential impact on public health strategies and reshaping the landscape of patient care. We find ourselves on the cusp of a transformative era by embracing holistic approaches and tailoring treatment paradigms to each individual. The insights gleaned from comprehending this nuanced interplay hold the potential to elevate liver health and substantially enhance the well-being of innumerable individuals across the globe.
